# Polling the Public to Select Flagship Species for Tourism and Conservation—A ‘Big Five’ for the Peruvian Amazon?

**DOI:** 10.1002/ece3.70983

**Published:** 2025-02-13

**Authors:** Maribel Recharte, Phyllis C. Lee, Sarah‐Jane Vick, Mark Bowler

**Affiliations:** ^1^ University of Stirling Stirling UK; ^2^ University of Suffolk Ipswich UK

**Keywords:** Amazonia, conservation marketing, ecotourism, flagship species, rainforest

## Abstract

Flagship species are used to promote conservation and tourism. Africa's famous ‘*Big five*’ have become marketing flagships that fundraisers and tourism promoters emulate globally. Species can be selected systematically for marketing using characteristics such as colour, size or behaviour, but this approach can overlook unique animals or homogenise selections. Alternatively, polling the public can reveal existing preferences for animals directly. We used questionnaires with tourists in the Peruvian Amazon to identify existing biases for species and rank them for suitability for tourism and conservation marketing. Polling revealed several species that would not be considered good flagship candidates using systematic methods based on species characteristics. ‘Free listing’ tourists expressed preferences at inconsistent taxonomic levels. The response ‘monkeys’ (infraorder Simiiformes) was highest ranked, followed by ‘jaguar’ ( *Panthera onca* ), ‘Amazon dolphin’ ( *Inia geoffrensis* ), ‘sloths’ (suborder Folivora), and ‘caiman’ (subfamily Caimaninae) and ‘birds’ (class Aves). When ranking from a preselected shortlist, jaguar, Amazon dolphins and sloths (represented by *Bradypus variegatus* ) remained popular, while vote splitting within higher taxonomic levels, in particular monkeys, made room for green‐winged macaw ( *Ara chloropterus* ) and anaconda ( *Eunectes murinus* ). When asked about their willingness to pay for excursions or donate to conservation, tourists were overwhelmingly more likely to quote larger figures for jaguars than any other species, but results for other species were more homogenous. Some popular taxonomic groups are diverse in Amazonia; up to 14 monkey species may be present at some sites Amazonia, alongside several hundred bird species. A *Big five* strategy obscures this diversity. Using physical characteristics as selection criteria underplays diversity and overlooks popular taxa—notably sloths for the Amazon. A strategy of polling the public to select popular species as flagships more directly identifies salient species for marketing and efficiently considers existing biases. However, diversity will trump a *Big five* approach in megadiverse areas.

## Graphical abstract

Flagship species are used to promote conservation and tourism, but selecting species purely for prescribed appealing characteristics may overlook unique flagships or homogenise selections. A strategy of polling the public can more directly identify existing preferences for species for marketing. Polling tourists in the Peruvian Amazon revealed that preferences for jaguars, Amazon dolphins and sloths are strong, but vote splitting within popular diverse groups like monkeys, leads to thier omission from Big Five rankings. In megadiverse areas, promoting diversity may trump the Big Five approach.

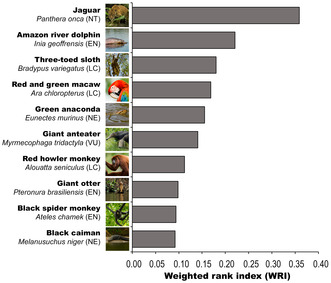

## 1 Introduction

Wildlife tourism has been widely examined for its potential to both enhance or damage nature conservation (D'Cruze et al. 2018; Wolf, Croft, and Green 2019; Meyer et al. 2021). While sustainability issues remain a concern (e.g., Vira and Adams 2009), tourism's indispensable role in financing global biodiversity conservation has been thrown into sharp relief by the local, national and international restrictions on travel during the 2020–2021 pandemic (Buckley 2021; Newsome 2021). To ensure the long‐term viability of tourism and understand how it impacts conservation, we first need to understand what wildlife tourists wish to see, what they are willing to ‘pay’ to see, and thus how they psychologically and economically value their viewing experiences of different wild species (Musa et al. 2021). Organisations raising funds for conservation, commercial tourism operators and government tourism boards have similar requirements for assessing species appeal and seek to harness these values to promote their activities and generate funds. One widespread mechanism for achieving this is the marketing of ‘flagship’ species. Flagship species are typically large charismatic vertebrates used to raise conservation awareness and public support, or to generate funds through wildlife tourism (Clucas, McHugh, and Caro 2008; Caro 2010). While key to the conservation and tourism strategies of NGOs and government bodies, conservation marketing using flagship species may also benefit private tourism companies and individuals. Wildlife tourism can give economic value to wildlife and landscapes, which is perceived as motivation for local stakeholders and policymakers to conserve them (Di Minin et al. 2013; Pegas et al. 2013). However, it should not be assumed that increasing tourism for these species will automatically benefit conservation. While tourists are more likely to pay to see, or donate for the conservation of, species that they find more appealing (e.g., Sekar, Weiss, and Dobson 2014; this study), the relationship between tourism and conservation is conflicted and contested. Nonetheless, marketing wildlife is therefore seen as a key strategy to conserving natural environments (Smith, Veríssimo, and MacMillan 2010; Veríssimo, MacMillan, and Smith 2011).

One of the classic examples of wildlife flagship marketing is the use of the ‘*Big five*’ of Southern and Eastern Africa; the lion ( *Panthera leo* ), leopard ( *Panthera pardus* ), buffalo ( *Syncerus caffer* ), elephant ( *Loxodonta africana* ) and rhinoceros ( *Diceros bicornis* and *Ceratotherium simum* ), around which perhaps the largest wildlife tourism industry is built (Walpole and Leader‐Williams 2002; Caro and Riggio 2013; Di Minin et al. 2013). The *Big five* were originally selected as big game hunting's most dangerous targets for hunting on foot, but now have an important socio‐economic value to wildlife tourism, bringing an enormous number of tourists to Southern and Eastern Africa (Caro and Riggio 2013), and finding their way into popular culture (e.g., Capstick 1977; Taylor, Hinde, and Du Toit 2005; Donaldson and Scheffler 2017). Because of the demand generated by the *Big five*, driven largely by the runaway marketing of these *six* species, some private game reserves in South Africa have re‐introduced these species to fulfil tourist and hunter expectations, the cost of which is estimated at between $97,500–1.8 million per private protected area (Sims‐Castley et al. 2005; Maciejewski and Kerley 2014).

Such is the success and draw of the *Big five* concept in Africa, various organisations have attempted to market *Big fives* for other countries, continents or ecosystems. Denali National Park, USA, proposed brown bear ( *Ursus arctos* ), wolf ( *Canis lupus* ), caribou ( *Rangifer tarandus* ), dall sheep ( *Ovis dalli* ) and moose ( *Alces alces* ) as their *Big five*, based on tourist satisfaction of sightings (Skibins et al. 2012). In Scotland, the Scottish Natural Heritage led a voting campaign to select the *Big five* for Scotland to drive more tourism to Scotland, eventually selecting the golden eagle ( *Aquila chrysaetos* ), harbour seal ( *Phoca vitulina* ), European otter ( *Lutra lutra* ), red deer ( *Cervus elaphus* ) and red squirrel ( *Sciurus vulgaris* ) (Scottish Natural Heritage 2015). Similarly, the IUCN (International Union for the Conservation of Nature) identified a *Big five* for Europe through public voting: lynx ( *Lynx lynx* and *Lynx pardinus* ), wolf ( *Canis lupus* ), brown bear ( *Ursus arctos* ), wolverine ( *Gulo gulo* ) and European bison ( *Bison bonasus* ) (IUCN 2014). In South America, WWF (World Wildlife Fund 2015) selected a *Big five* for the Cerrado savannah and Pantanal wetland; jaguar ( *Panthera onca* ), giant armadillo ( *Priodontes maximus* ), lowland tapir ( *Tapirus terrestris* ), giant anteater ( *Myrmecophaga tridactyla* ) and maned wolf ( *Chrysocyon brachyurus* ), while the tourist board for Madre de Dios, Peru, promoted an Amazonian *Big five* on billboards in Peru: jaguar (*Pantera onca*), giant otter ( *Pteronura brasiliensis* ), black caiman ( *Melanosuchus niger* ), Andean cock‐of‐the‐rock ( *Rupicola peruvianus* ) and the green‐winged macaw ( *Ara chloropterus* ) (Personal observation 2018).

Regardless of what the *Big five* might look like for any region, the identification of suitable species to act as the focus of marketing campaigns is considered important to conservation and wildlife tourism (Veríssimo, MacMillan, and Smith 2011). While it is important to understand the species traits that influence visitor preferences, for example, danger in the case of the original Big Five, or unique behaviours, conservation status, endemicity, body size or weight (Santarém et al. 2019), it is also pertinent to assess whether preferences exist using general tourist responses. More detail might be achieved by investigating the tourists ‘*willingness to pay*’ for tours, a measure used to predict the amount of money that a person is willing to pay for goods or services (Chung et al. 2011; Adamu et al. 2015) that has been applied to wildlife tourism (e.g., White, Bennett, and Hayes 2001; Sekar, Weiss, and Dobson 2014). Similarly, ‘*Willingness to donate’* measures people's potential to contribute to wildlife conservation without receiving anything in return (Meer, Badza, and Ndhlovu 2016) and has been used in the conservation of game reserves (Adamu et al. 2015), endangered species (Lindsey et al. 2007; Richardson and Loomis 2009) and for flagship species selection on a local level (Di Minin et al. 2013; White et al. 1997).

Travel in South America is considered greatly underdeveloped, receiving a disproportionately small proportion of international travellers (de Oliveira Santos 2015), but consequently is one of the fastest‐growing regions for the industry (Navarro‐Drazich 2024). Within the continent, tourism focusses on mountainous regions in the West, and Coastal regions in the East, leaving the largest tropical forest on Earth sparsely visited (Navarro‐Drazich 2024). The Amazon rainforest ecosystem is biodiverse and has iconic appeal, but wildlife tourism is on a smaller scale relative to many African *Big five* safari destinations (Vidal, Paim, and Mamede 2023). There is a rich megafauna, with many flagship species candidates, but marketing is currently centred around a few species, notably the seldom‐seen jaguar (Vidal, Paim, and Mamede 2023). The relative importance of the Amazon's large animal species to tourists and their potential to generate funds for conservation have been little explored, and the practice of using simple polls to identify flagship species has not been examined. We use interviews with tourists in the Peruvian Amazon to identify existing preferences for species that might be most suitable as flagship species for the region. We address whether potential candidates could be determined from existing tourist preferences, rather than attempting to select ‘optimal’ flagship species based on characteristics associated with ‘appeal’. We assess the relative value of these species by asking which tourists would most like to see, and use *willingness to pay to see* and *willingness to donate* as a further measure of the relative appeal of different species as flagships. We consider the suitability of species‐driven marketing approaches for enhancing conservation and tourism and review the method by which flagships are selected.

## 2 Methods

### 2.1 Study Locations

Between May 2015 and April 2016, we approached tourists in travel hub cities of the two most‐visited regions of the Peruvian Amazon: Loreto in the north and Madre de Dios in the south. We were unable to gain permission to airports to recruit participants, and early pilot studies revealed that using hotels biased samples towards tourists of a similar economic backgrounds, and even groups travelling on the same package. However, in Iquitos, the main city in Loreto, tourists from a greater variety of backgrounds could be sampled at visitor attractions. We included the two most‐visited attractions in the city: *Pilpintuwasi Amazon Animal Orphanage* and *Centro de Rescate Amazonico* CREA (Amazon Rescue Centre). Because these were both animal centres, (we also approached the most‐visited nonanimal attraction at the time: 3) *Museo de Culturas Indigenas Amazonicas* (Museum of Indigenous Amazonian Cultures). Attractions in the city of Puerto Maldonado, were not as widely visited, but we were able to sample at a transport hub managed by *Rainforest Expeditions*, where tourists await fluvial transfer to three different lodges. Although experiences at the lodges overlapped in nature, each catered for different tourist interests (*Personal Communication*, Rainforest Expeditions Inc.). Remote lodges serviced more clients with special wildlife interests, while lodges closer to the city frequently hosted clients on the rainforest leg of a more general tour package. This transport hub allowed us to survey as representative a range of interest groups as practically possible.

While two of our sites were animal attractions, globally, zoo visitors come from a wide range of backgrounds and interests (Roe and McConney 2015). We tested this assumption by asking visitors to score their motivations for travel at the start of the interviews. We compared responses across participants recruited at different locations to determine whether the sample was representative of those visiting the region.

### 2.2 Questionnaire

A questionnaire designed to test for existing preferences for Amazonian animal species was piloted with 30 participants, including both English and Spanish speakers to ensure that all the questions were clearly understood. After these pilot interviews, some minor changes in wording were made, and the sampling strategy was established.

We used a questionnaire including fixed‐response and open‐ended questions administered on an electronic survey platform: ‘Qualtrics’ (Qualtrics 2019). Tourists were approached with a tablet at venues. Only adults over 18 years old were interviewed. Fixed response questions were answered by clicking on options for ranking questions and sliding scales for assigning Likert‐scale (Likert 1932) or monetary values. Although the Qualtrics interface cannot be reproduced here, the questions are reproduced in Table 1.

**TABLE 1:** Main questions asked of tourists in the Peruvian Amazon. *‘Tourists with experience’*: People that had already visited the forest in Amazonia either on their current trip or on a previous visit, *‘Tourists with no experience’*: People that had not yet visited Amazonian forests. Underlining and italics were used to improve understanding of the questions. The photos referred to in section B. are reproduced in Figure 1.

Type of question	Question
Open‐ended Interviews were terminated if the respondent was under 18.	Age
Open‐ended	Gender
Open‐ended	Home country
Fixed response	Motivation for travel Likert‐scale (Likert 1932) slider to assign importance on a scale from 0 (not a factor) to 5 (of high importance) for the categories: Biodiversity, Culture, Adventure, Landscape.
Fixed response *This question categorised the respondent as with or without experience of the forest and determined which versions of the questions they saw subsequently*.	Have you visited the Amazonian rainforest, either on this trip or in the past?
Open‐ended *For ‘tourists without experience’*	A.1. What animals would you most like to see on a trip to the Amazon rainforest? List up to 5, with the most desirable first.
Open‐ended *For ‘tourists with experience’*	A.2. What animals did you most like seeing on your trip(s)? List up to 5 with the most desirable first.
Open‐ended *For ‘tourists with experience’*	A.3. What animals, that you did not see, would you have most liked to see on your trip(s)? List up to 5 with the most desirable first.
Fixed response *For all respondents*	B. From the photos below, please rank the five animals that you would most like to see on a trip to the Amazon Rainforest (selecting the most desirable first).
Fixed response *For all respondents*	C.1. Willingness to pay: ‘If you were already in the Amazon rainforest, how much would you be prepared to spend on a single day excursion to see the following animals? (For a separate trip to see only the animal mentioned). Indicate within the range of $1–$1000 American dollars.
Fixed response *For all respondents*	C.2 Willingness to donate ‘how much would you be prepared to give as a donation towards the conservation of each of these species? For each species, if prepared to donate, indicate within the range of $1–$100 American dollars.)

To allow us to characterise the sample, and screen for biases, we asked respondents for their age, gender and home country. The interviewees came from socioeconomic groups that were already travelling to the region so represented our target population, including both domestic and international visitors to the region. We were unable to ask socioeconomic questions of tourists at our host venues, but we selected our sites to include tourists from a cross section of budgets. To screen for biases in different interests and motivation for travel between tourists approached at different venues, we asked participants to score their motivation to travel in the broad categories of ‘biodiversity’, ‘culture’, ‘adventure’ and ‘landscape’.

A total of 502 people were interviewed but 65 interviews were not completed, so we used 437 completed interviews for analysis. Sample sizes at the survey locations were as follows: *Pilpintuwasi Amazon Animal Orphanage*, *N* = 123 tourists, 28.1%, CREA Amazon Rescue Centre (*N* = 113 tourists, 25.9%), Museum of Indigenous Amazonian Cultures, (*N* = 106 tourists, 24.3%), Puerto Maldonado (*N* = 74 tourists, 16.9%). A further 21 (4.8%) questionnaires were completed with tourists at the three named sites in Loreto, but the survey location was not correctly recorded in Qualtrics. These were not used in analyses that used the venue as a parameter, but were included for other analyses.

All questions were written in English and administered in either English or Spanish, translated by the bilingual person administering the survey where necessary. Participation was voluntary, and participants were informed that their responses would be anonymised and that they could withdraw their consent at any time. Questionnaires and modes of administration were approved by the University of Stirling Research Ethics Committee.

### 2.3 Open‐Ended Questions

To frame the question in an appropriate way for each participant, the questionnaire first divided respondents into two groups; (1) people that had already visited the forest in Amazonia either on their current trip or on a previous visit, identified as *‘Tourists with experience’* and (2) people that had not yet visited Amazonian forests *‘Tourists with no experience’*.

*‘Tourists with no experience’* were asked one open‐ended question; A.1. ‘what species would you most like to see on a trip to the Amazon Rainforest?’ *Tourists with experience’* were first asked two open‐ended questions; A.2 what animals they had most liked seeing on their trip to the Amazon rainforest, and A.3 what animals *that they did not see* would they most liked to have seen (Table 1).

### 2.4 Fixed Response Questions

All respondents were presented with images and common names for 21 wildlife species, in a 3 by 7 grid, arranged in a randomised order for each participant, and asked to rank the top five they would like to see on a trip to the Amazon rainforest, using a 5‐point scale from ‘most desirable (1)’ to ‘fifth most desirable (5)’. Wildlife species were selected for inclusion based on published interviews made in the Pacaya‐Samiria National Reserve (PSNR) (Recharte, Bride, and Bowler 2015) in which we asked local people which animals they thought tourists would like to see in the rainforest. To ensure we did not leave out key species from the south of Peru, we also reviewed the Peruvian Tourist Board marketing in posters and leaflets at travel hubs in Peru for frequently mentioned species. This led to the inclusion of one additional species, the Andean cock‐of‐the‐rock ( *Rupicola peruvianus* ). As far as possible, images were selected and cropped to produce an evenly lit, tight crop of the whole animal or a three quarters view in a neutral, static position, to represent the animals as consistently as possible and minimising the surroundings shown around them. Images of obviously captive animals were not used.

Species included were as follows: Jaguar *Onca Panthera*, Amazon river dolphin *lnia geoffrensis*, Three‐toed sloth *Bradypus variegatus*, Red and green macaw *Ara chloropterus* , Green anaconda *Eunectes murinus*, Giant anteater *Myrmecophaga tridactyla*, Red howler monkey *Alouatta seniculus*, Giant otter *Pteronura brasiliensis*, Black spider monkey *Atetes chamek*, Black caiman *Melanusuchus niger*, Harpy eagle *Harpia harpyja*, Common squirrel monkey *Saimiri Sciurus*, Tucuxi dolphin *Sotalia fluvialis*, Capybara *Hydrochoerus hydrochaeris*, Lowland tapir *Tapirus terrestris*, Andean cock‐of‐the‐rock *Rupicota peruvianus*, Neotropical otter *Lontra longicaudis*, Bald uakari monkey *Cacajao calvus*, Common caiman *Caiman crocodilus*, Brown capuchin *Sapajus macrocephalus*, White‐lipped peccary *Tayassu pecari*.

Participants were also asked about their *willingness to pay* and *willingness to donate* for a subset of 11 of these species, selected using the same criteria, but narrowing the list to the most salient half from Recharte, Bride, and Bowler (2015) to keep the questionnaire manageable. These measures were used to quantify the relative popularity and potential of species as flagships for the tourist industry or conservation following Van der Meer, Badza, and Ndhlovu (2016) rather than as a means of estimating the actual economic value of the species as the concept of *willingness to pay* is more commonly used (Abdeta 2022). First, to explore the popularity of a species using *willingness to pay*, we allowed respondents to use a sliding bar to decide the amount of money they would be willing to spend on a single day trip to see this animal. Second, we asked about *willingness to donate* for the conservation of specific animal; again, a sliding bar was used to explore their preference for species and donation amount. Of course, all respondents were already in the position of paying for some tourism experiences, and our questions were framed hypothetically as ‘in addition to’ the experience they were undergoing at the time.

### 2.5 Data Analysis

Descriptive statistics and correlations were calculated using Statistical Package for Social Science (SPSS IBMcorp.) version 21.0 for Windows.

Scores for participants' motivations to travel were converted to ranks, and the importance of biodiversity as a motivation for travel was compared between samples collected at different venues using a Kruskal–Wallis test.

Responses on preferences for animal taxa were converted into binomial categories for analysis. Since respondents could give different numbers of responses in open‐ended questions, we used the order of responses to determine the most salient (Quinlan 2005).

We calculated the mean rank of the answers for animal preferences using a Weighted Rank Index (WRI) to standardise the answers with a mean value, the index was calculated separately for open‐ended questions (A.1, A.2, A.3) and closed questions (B) (Nepal and Weber 1993; Gillingham and Lee 2003), where:

$$\mathrm{WRI}=\sum_{i}^{n}\left(\frac{1}{Rᵢ}\right)/N$$

*n* = number of respondents ranking species, *Rᵢ =* rank of the *ith* order, *N =* total number of respondents in the sample.

## 3 Results

A total of 437 tourists completed the survey, 55% female and 45% male, with a mean age of 39.7 years (SD 14.5, range 18–88). Tourists sampled came from 40 different home countries. Nineteen were domestic tourists from other parts of Peru, while the most‐represented foreign nations were as follows: the United States (68), Germany (41), Chile (32), the United Kingdom (21) and France (18). The broad international spread reflects tourism figures for Peru (MINCETUR 2018), but is a more targeted sample of those visiting Amazonian regions.

There were no differences between respondents recruited at different sampling locations in the priority they gave to biodiversity in their motivation to travel (Kruskal–Wallis test, H = 6.6085 (3, *N* = 326), *p* = 0.085). With the assumption that no major groups or types of tourists were excluded by our sampling strategy, this suggests that we recruited participants with motivations for travel that are broadly representative of those visiting the region.

### 3.1 Most Salient Animals

In the subgroup ‘*tourists with no experience’* (*n* = 329), 299 people answered the question ‘What animals would you *most like to see* on a trip to the Amazon Rainforest?’ From the subgroup ‘*tourists with experience’* (*n* = 108), 90 answered the question ‘What animals did you *most like seeing* on your trip(s)?’ and 94 responded to the question ‘What animals, that you *did not see*, would you have most liked to see on your trip?’

Responses did not consistently refer to species, genera or other taxonomic levels. For example, different respondents may have responded ‘scarlet macaw’, ‘macaws’, ‘parrots’ or ‘birds’. So, we calculated the WRI for ‘responses’, which could refer to species, genera, families or orders, or paraphyletic groups of animals, such as ‘birds‐of‐prey’. As such, responses represented the most preferred, salient or memorable animal ‘categories’ rather than mutually exclusive Linnean taxonomic units.

For tourists who had not yet been to the forest, there were 57 different responses to the question; ‘What animals would you most like to see on a trip to the Amazon Rainforest?’ ‘Monkeys’ as an infraorder (Simiiformes) were highest ranked, followed by an individual species; the jaguar ( *Panthera onca* ), a suborder, sloths (Folivora), a class, birds (Aves) and another suborder, caiman (Caimaninae) (Table 2). No individual monkey species or genus made the top 20.

**TABLE 2:** The most salient animals ‘free listed’ by tourists in the Peruvian Amazon in response to open‐ended questions on the animals they would most like to see or have enjoyed seeing. Answers were not restricted by taxonomic levels and were not mutually exclusive. Thus, the results are ranked preferences for preferred, salient or memorable animal ‘categories’ rather than Linnean taxonomies.

Free listing response	Taxonomic level		Tourists who had not yet visited the rainforest	Tourists who had already visited the Amazon rainforest	
			‘What animals would you most like to see on a trip to the Amazon rainforest?’	‘What animals did you most like seeing on your trip?	‘What animals, *that you did not see*, would you most liked to have seen on your trip?
			Ranking	Ranking	Ranking
Monkeys	Order	Simiiformes	1	1	2
Jaguar	Species	*Panthera onca*	2	5	1
Sloths	Suborder	Folivora	3	3	10
Birds	Class	Aves	4	2	8
Caiman	Subfamily	Caimaninae	5	10	3
Macaws	Tribe	Arini	6	7	16
Amazon river dolphin	Species	*Inia geoffrensis*	7	4	4
Anaconda	Genus	Eunectes	8	17	6
Dolphins	Infraorder	Cetacea	9	38	5
Snakes	Suborder	Serpentes	10	8	12
Manatee	Genus	Trichechus	11	6	7
Butterflies	Suborder	Rhopalocera	12	9	27

There were 68 different responses to the question; ‘What animals did you most like seeing on your trip to the Amazon Rainforest?’ The response ‘monkey’ was ranked highest, followed by ‘birds’, ‘sloth’, ‘Amazon river dolphin’ and ‘jaguar’ (Table 2). One named monkey species (or genus depending on the taxonomy used) ‘woolly monkey’ ( *Lagothrix lagotricha* or *Lagothrix* spp.) was also ranked in the top 20.

There were 56 different responses to the question; ‘What animals *that you did not see*, would you have most liked to have seen on your trip to the Amazon Rainforest?’ The response ‘jaguar’ was ranked highest, followed by ‘monkeys’, ‘caiman’, ‘Amazon river dolphin’ and ‘dolphins’ (Table 2).

### 3.2 The Most Desirable Species in Amazonia

**FIGURE 1:**
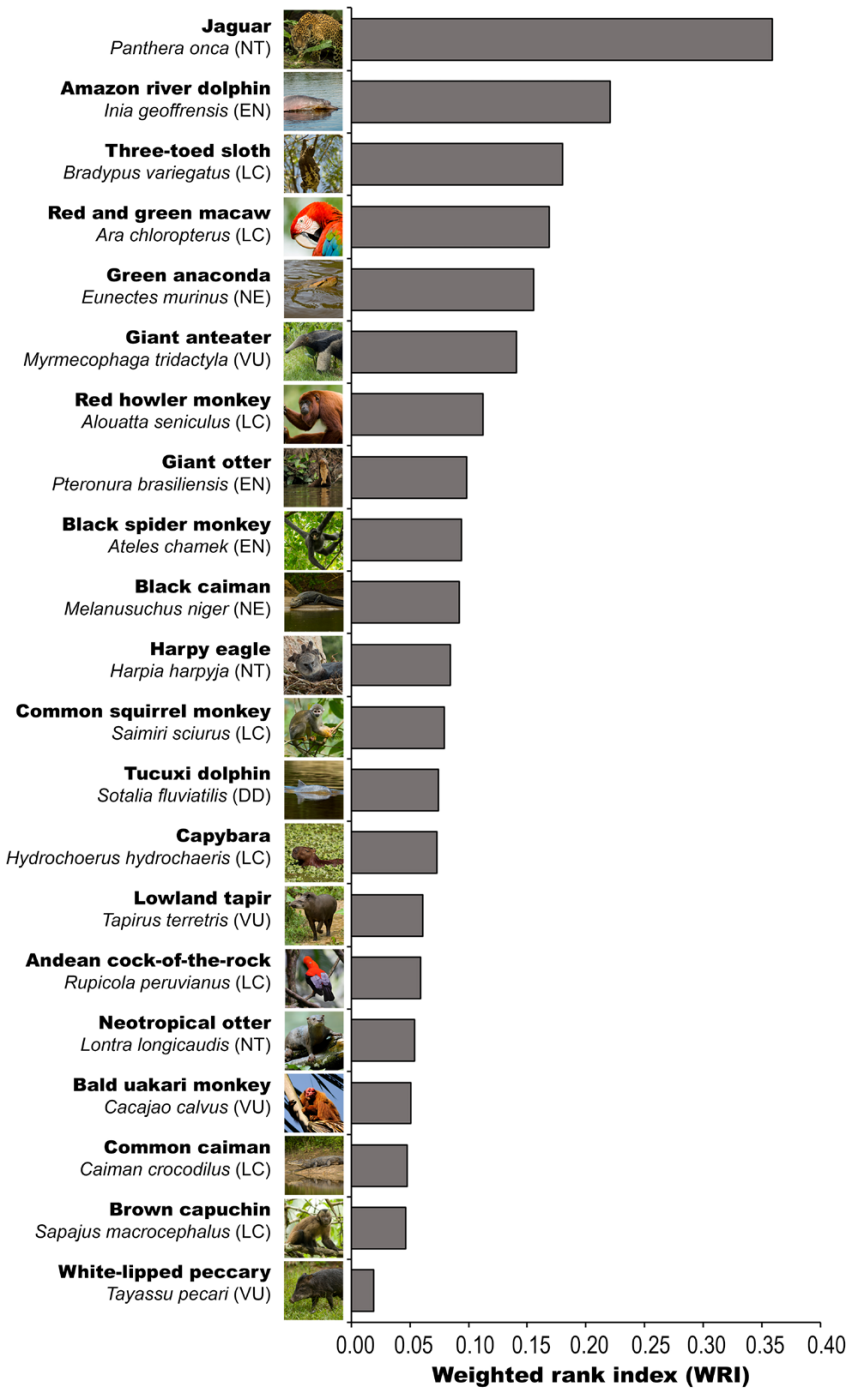
The ‘most wanted’ animals to see in Amazonia selected by tourists in the Peruvian Amazon from a shortlist of twenty‐one pictures. Ranked using the Weighted rank index (Nepal and Weber 1993). Note that in diverse groups, such as the monkeys, vote splitting between species may be apparent. IUCN Red List categories (not presented in the questionnaire): DD = Data deficient, EN = Endangered, LC = Least concern, NE = Not evaluated (IUCN 2023), NT = Near threatened, VU = Vulnerable. (Picture credits: Harpy eagle: Diego Balbuena; Cock‐of‐the‐rock Bill Bouton; all others by the authors).

When asked to rank the animals they would most like to see in Amazonia from the shortlist of 21 pictures, the top five‐rated animals using the WRI, were jaguar, Amazon river dolphin, three‐toed sloth, green‐winged macaw and anaconda (Figure 1). Some taxonomic groups are diverse and were represented by several species in the 21 shortlisted animals. For example, five primates were included. These may have a lower WRI because votes were split between several species.

### 3.3 Willingness to Pay for Potential Flagship Species

Given that the questions were somewhat abstract, in that respondents did not have to pay the amounts they selected, we used the ‘willingness to pay’ and ‘willingness to donate’ scores to determine the *relative* value and potential of different species for tourism marketing, rather than to determine actual prices that could be achieved for these hypothetical tours and marketing campaigns.

Of 437 tourists, 363 (84%) were willing to pay to see one or more of the animals listed. Over two‐thirds of tourists (68%) were willing to pay to see jaguar, some indicating that they would pay a maximum price of $1000 US dollars (the upper limit of the sliding scale) to guarantee sightings of one, with $159 dollars as a mean value for all the tourist that selected nonzero values for this animal. A majority of tourists also indicated they would pay extra to see Amazon river dolphins (64%, mean $102.86), spider monkey (54%, mean $93.85), black caiman (54%, mean $92.15), green‐winged macaw (54%, mean $83.05), harpy eagle (51%, mean $97.11), uakari monkey (51%, mean $91.54) and giant otter (51%, mean = $88.83).

Of all the interviewees, 338 (78%) were willing to donate for the conservation of one or more animal from the list. More than 50% of tourists were willing to donate for the conservation of three specific animals in Amazonia: the jaguar (64%), Amazon river dolphin (62%) and giant otter (51%). However, differences were not pronounced, with around half of people indicating they would be prepared to donate for the conservation of any species.

**FIGURE 2:**
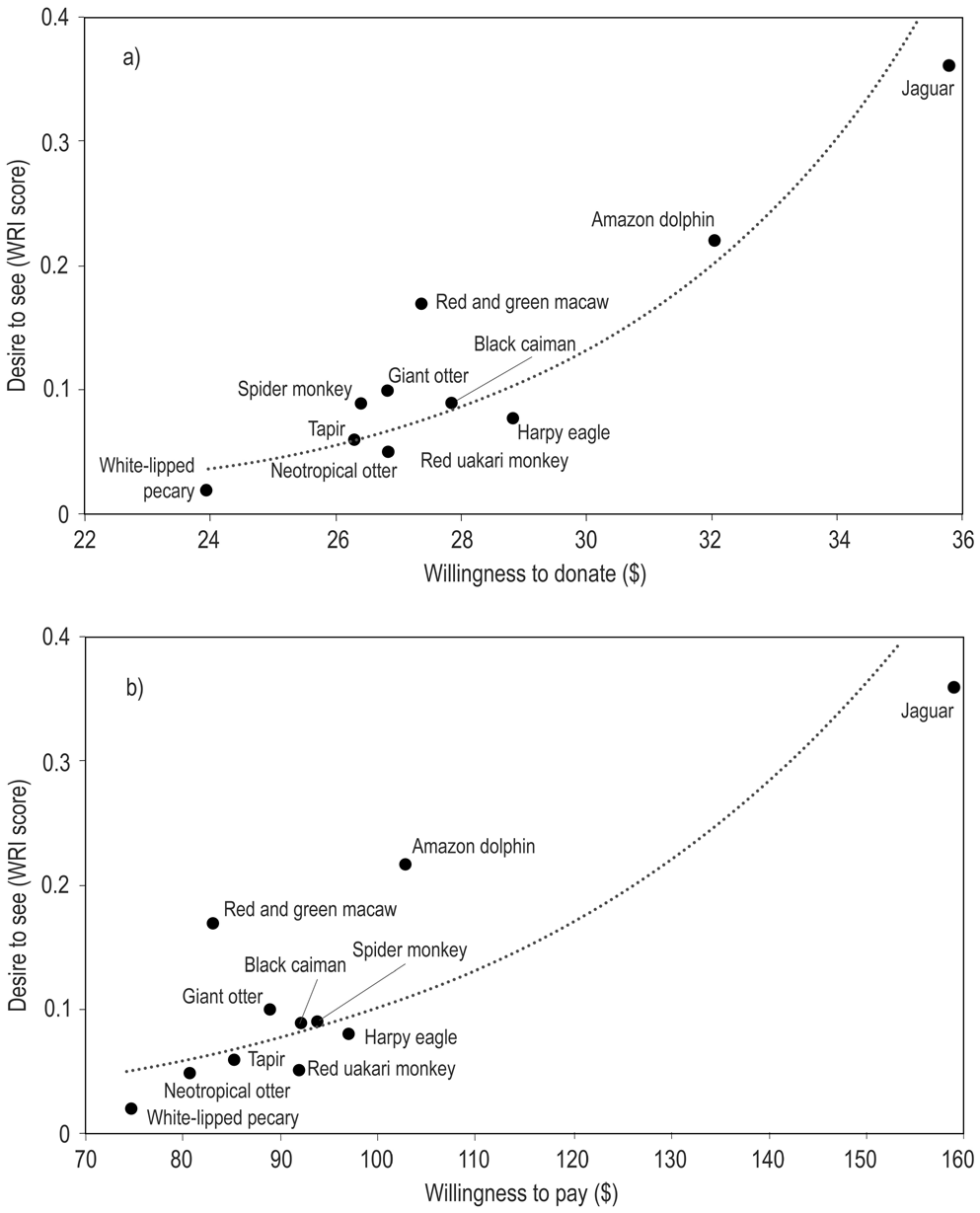
The relationship between Amazonian animals that tourists would most like to see (ranked using the Weighted rank index, Nepal and Weber 1993), and their *willingness to pay* ($ USD) to see them or *donate* for their conservation.

The desirability of species, as established by ranking from images in previous questions, was more strongly related to *willingness to donate* than to *willingness to pay* to see species (Figure 2). Several species, notably green‐winged macaws and Amazon river dolphins, are easily seen on a daily basis at many sites, without paying extra for excursions, which may explain this pattern.

## 4 Discussion

Our study does not set out to determine how or why species are popular. Historical and cultural biases, rarity and availability of information are intertwined with the effects of physical characteristics like colour and size (Bowen‐Jones and Entwistle 2002). Separating these factors is problematic and needs to account for phylogenetic relationships and uniqueness (Veríssimo, MacMillan, and Smith 2011), but the results of these, in the form of biases or preferences, are there to use, irrespective of how they were derived. In practice, conservation and ecotourism marketing campaigns that set out to select flagships on appeal, without consulting relevant public groups, often fail to consider an adequate range of species or characteristics in a systematic way (Smith et al. 2012). While comprehensive systematic approaches to do this have been developed (e.g., Santarém et al. 2019; Lundberg et al. 2020), accounts of them being used are rare. Meanwhile, an alternative approach of polling the public has been more widely used, but not studied (e.g., Skibins et al. 2012; Scottish Natural Heritage 2015; IUCN 2014; World Wildlife Fund 2015). Polling will produce a list that considers existing biases alongside physical appeal and may be a more efficient approach, especially where funding is not available to promote appealing, but lesser‐known, potential flagship species. We replicated this alternative approach for an area where there is a high diversity of potential flagship species, but where only a few species are consistently used in tourism marketing campaigns.

In our survey, one species, the jaguar, clearly emerged as the most salient and desirable species for Amazonian wildlife tourists. This is not an unexpected result: In Africa, tourists also have marked preferences for large carnivores; leopards, lions and cheetahs, and willingness to pay to see these is higher than for other species (Lindsey et al. 2007; Di Minin et al. 2013; Meer, Badza, and Ndhlovu 2016). The big cats have several features identified as appealing to people; they are large predators embodying a genuine potential threat to humans, are included in the IUCN's Red List of Threatened Species, have forward‐facing eyes, facial markings and some have bright colouration (Macdonald et al. 2015). There are other felids in Amazonia: puma ( *Puma concolor* ), ocelot ( *Leopardus pardalis* ), margay ( *Leopardus wiedii* ) and jaguarundi (*Herpailurus yagouaroundi*), but none compete with the jaguar for salience and tourist preference. While the size of jaguar sets it above other South American felids in these criteria, the jaguar also has a long history of coverage in popular culture and is an iconic species said to evoke Amazonia itself (Rabinowitz 2000). Similarly, the Amazon river dolphin, the second most popular single species to tourists in our study, is embedded in Amazonian culture (Montgomery 2009), and the popularity of watching dolphins in the wild is a global phenomenon (O'Connor et al. 2009). This history of culture and media coverage is hard to separate from the effects of targeted marketing or the suitability of species based on their appealing characteristics.

While Jaguars and Amazon river dolphins were popular choices, tourists were generally unable to name many species during free listing. Preferences referred to a wide range of taxonomic levels, and the most salient or desirable animals did not fall into consistent taxonomic groups when open‐ended questions were asked. ‘Monkeys’ as a superorder (Ceboidea) were highest ranked, followed by the individual species jaguar ( *Panthera onca* ) and Amazon river dolphin ( *Inia geoffrensis* ), a suborder, sloths (Folivora), a class, birds (Aves) and another suborder Caiman (Caimaninae). Few tourists were specific in their choices, giving the impression that ‘any monkey will do’ for marketing purposes. In the *Big five* marketing model, there is a precedent for combining more than one species. The African *Big five* includes two rhinoceros species as a single flagship, despite them belonging to different genera. In the case of Amazonian monkeys, there are more than 50 species in Peru alone, and individual sites will typically have 8–13 species (Aquino et al. 2015). While single‐species campaigns for primates have successfully generated tourism, funding and support for conservation (e.g., Xiang et al. 2011; Abondano et al. 2023), a *Big five* approach that selects a single representative monkey would fail to recognise a large number of charismatic and threatened species. This would be at odds with alternative marketing approaches that aim to leverage the diversity of primates and challenge tourists to see as many species as they can—an experience more akin to birdwatching than a *Big five* safari (Mittermeier 2016).

The pressing need for the conservation of primates, combined with their high visibility and potential for conservation‐sensitive tourism (Hansen et al. 2023) may call for a strategy that harnesses existing preferences for monkeys, and also takes advantage of the diversity in the group in a way that the Big five model cannot. It is apparent that tourism marketing in the Amazon differs from that of Africa; species are generally less recognisable, even to tourists in situ in the Amazon. The issue of familiarity is central to our results. Species that are well‐known scored highly, while lesser‐known, potentially excellent flagships did not. The Amazon's rare dog species, the bush dog ( *Speothos venaticus* ) and the short‐eared dog ( *Atelocynus microtis* ) are not well known to public, were never mentioned in free listing, and did not make the shortlist for our follow‐up question. However, according to the criteria suggested for flagship species, such as rarity, large size and forward‐facing eyes (Smith et al. 2012; Macdonald et al. 2015), they are very well suited as flagships. Similarly, the giant otter ( *Pteronura brasiliensis* ), fulfils all the criteria of large size and appeal, and is considered very important for tourism in parts of its range (Tomas et al. 2015; Groenendijk 2019). As a result, it is already marketed for tourism and used as a flagship species (Kruuk 2006). However, giant otters were not particularly well known by the tourists we interviewed, or highly ranked for ‘desire to see’. In practice, selection of a species for marketing at the point of sale may hinge as much on necessity as suitability; the ease of viewing species like giant otters at some sites makes them a focal point for tours, and marketing a species that tourists are likely to see may lead to increased levels of satisfaction (Torres‐Sovero et al. 2012). Nonetheless, our survey highlights potential discrepancies between the perceptions of conservation and tourism practitioners in the field, and the actual salience and preferences for species by tourists.

It may not always be not clear why some species are more, or less popular than expected, based on their characteristics (Macdonald et al. 2015), or their prevalence in marketing material. The recent ‘rise to fame’ of the sloths is an example of a species being rapidly elevated to a tourism and conservation flagship. Sloths, consisting of two distantly related genera (*Bradypus* and *Choloepus*), are now clear contenders for the Amazonian *Big five*. However, the popularity of sloths is a relatively new phenomenon. A series of highly popular viral internet videos, followed by network television series and feature films, propelled sloths to cult status as wildlife flagships (e.g., Animal Planet 2013).

Macaw (Subfamily Arinae) tourism in Tambopata also received a chance boost from the media, this time in the 1990s as a result of high‐profile science journalism (Munn 1994). Macaws meet the criteria for charismatic species (Veríssimo, MacMillan, and Smith 2011; Lišková, Landová, and Frynta 2015) and have long been in the public conscious. Furthermore, across Amazonia, they visit clay licks in large numbers to eat soil to obtain scarce minerals, creating spectacular wildlife viewing opportunities. Sightings are guaranteed and tourist satisfaction high (Torres‐Sovero et al. 2012). When National Geographic (Munn 1994) covered scientific research on the phenomenon, tourism around clay licks developed rapidly, and has generated high volumes of tourism over many years, benefiting local communities and influencing the management of the river basin (Torres‐Sovero et al. 2012).

High profile and viral media phenomena that bring species into the public eye and inspire tourism are hard to predict. The sloth and macaw media coverage were not driven by tourism marketing but have subsequently been used by the industry. We can attempt to identify which species are most suitable for marketing, based on physical characteristics (Santarém et al. 2019), but in our surveys, favoured traits were not always present in popular species. The appeal of sloths does not appear to be based on the possession of any of the traits deemed appealing in flagship species research (Macdonald et al. 2015).

In keeping with the original African *Big five*, some popular species in our survey are imposingly large and surrounded by a perception of danger. Caiman and anaconda share these traits but have few other characteristics recognised as important for flagships. Species representing such ‘attractive threats’ may be expected to polarise opinion in a target audience or induce negative responses for local people in the destination country, just as the *Big five* do in Africa (Gillingham and Lee 2003). As a result, they may not be selected for marketing campaigns (Schlegel and Rupf 2010). Alternatively, they may become destinations in themselves (e.g., diving with great white sharks in South Africa). However, such activities with large or dangerous species may be questionable with regard to species conservation as opposed to simple revenue generation for tourist operators. The value of a “*Big five*” campaign to species conservation needs to consider the complexities and trade‐offs involved in inducing or mitigating conservation conflicts in the target country (Western, Waithaka, and Kamanga 2015).

While using physical characteristics to identify potential flagships is potentially useful (e.g., Santarém et al. 2019), unless the capacity to promote these species on a large scale is present, this may not always be the most efficient approach. Existing biases and chance large‐scale media successes may be hard to match through marketing, and local campaigns may do better to select and market products using species that are already popular or at least recognisable for potential clients. As such, the widespread approach taken by tourist boards and NGOs of polling the public to select taxa for a park or region is appropriate and useful. Selecting the top five of these has clear advantages in simplifying the facility for destination marketing campaigns and may benefit from the target audience's familiarity with the *Big five* concept. However, when we try to construct a *Big five* for Amazonia, it becomes apparent that the format is too restrictive. The jaguar is far and away the most appealing flagship and biggest draw for tourists, but a diversity of other appealing animals would be excluded by a *Big five* approach. Furthermore, the diversity itself, particularly of highly regarded groups like the monkeys, may represent a more powerful marketing tool, and a more fittingly diverse marketing strategy might be more appropriate in megadiverse regions like this.

## Author Contributions

**Maribel Recharte:** conceptualization (lead), data curation (lead), formal analysis (lead), investigation (lead), methodology (lead), project administration (lead), resources (equal), writing – original draft (lead), writing – review and editing (supporting). **Phyllis C. Lee:** formal analysis (supporting), methodology (supporting), supervision (lead), writing – original draft (supporting), writing – review and editing (supporting). **Sarah‐Jane Vick:** supervision (supporting). **Mark Bowler:** conceptualization, investigation (supporting), methodology (supporting), supervision (supporting), visualization (equal), writing – original draft (supporting), writing – review and editing (lead).

## Ethics Statement

All research complied with the journal's Code of Conduct for authors contributing articles and had ethical clearance from the University of Stirling's ethics committee, and informed consent from all participants.

## Conflicts of Interest

The authors declare no conflicts of interest.

## Supporting information


Appendix S1.

## Data Availability

The data that support the findings of this study are available as a Supporting Information to this manuscript.
